# Efficacy of the combination of monoclonal antibodies against the SARS-CoV-2 Beta and Delta variants

**DOI:** 10.1371/journal.pone.0284173

**Published:** 2023-05-04

**Authors:** Chatikorn Boonkrai, Thomas S. Cotrone, Watchadaporn Chaisuriyong, Terapong Tantawichien, Usa Thisyakorn, Stefan Fernandez, Taweewun Hunsawong, Matthew Reed, Tossapon Wongtangprasert, Thittaya Audomsun, Tanapati Phakham, Chadaporn Attakitbancha, Pijitra Saelao, Dorota Focht, Raymond Kimbung, Martin Welin, Aijaz Ahmad Malik, Trairak Pisitkun, Nattachai Srisawat

**Affiliations:** 1 Interdisciplinary Program of Biomedical Sciences, Graduate School, Chulalongkorn University, Bangkok, Thailand; 2 Faculty of Medicine, Center of Excellence in Systems Biology, Chulalongkorn University, Bangkok, Thailand; 3 Department of Virology, Armed Forces Research Institute of Medical Sciences (AFRIMS), Bangkok, Thailand; 4 Excellence Center for Critical Care Nephrology, King Chulalongkorn Memorial Hospital, Bangkok, Thailand; 5 Faculty of Medicine, Center of Excellence in Critical Care Nephrology, Chulalongkorn University, Bangkok, Thailand; 6 Tropical Medicine Cluster, Chulalongkorn University, Bangkok, Thailand; 7 Faculty of Medicine, Department of Medicine, Division of Infectious Diseases, Chulalongkorn University, Bangkok, Thailand; 8 The Excellence Chulalongkorn Comprehensive Cancer Center, King Chulalongkorn Memorial Hospital, Bangkok, Thailand; 9 SARomics Biostructures AB, Medicon Village, Lund, Sweden; 10 Faculty of Medicine, Center of Excellence in Computational Molecular Biology, Chulalongkorn University, Bangkok, Thailand; 11 Faculty of Medicine, Department of Medicine, Division of Nephrology, King Chulalongkorn Memorial Hospital, Bangkok, Thailand; 12 Department of Critical Care Medicine, Center for Critical Care Nephrology, The CRISMA Center, University of Pittsburgh, School of Medicine, Pittsburgh, PA, United States of America; 13 Academy of Science, Royal Society of Thailand, Bangkok, Thailand; St Jude Children’s Research Hospital, UNITED STATES

## Abstract

The pandemic of severe acute respiratory syndrome coronavirus 2 (SARS-CoV-2) is currently the biggest healthcare issue worldwide. This study aimed to develop a monoclonal antibody against SARS-CoV-2 from B cells of recovered COVID-19 patients, which might have beneficial therapeutic purposes for COVID-19 patients. We successfully generated human monoclonal antibodies (hmAbs) against the receptor binding domain (RBD) protein of SARS-CoV-2 using developed hybridoma technology. The isolated hmAbs against the RBD protein (wild-type) showed high binding activity and neutralized the interaction between the RBD and the cellular receptor angiotensin-converting enzyme 2 (ACE2) protein. Epitope binning and crystallography results displayed target epitopes of these antibodies in distinct regions beneficial in the mix as a cocktail. The 3D2 binds to conserved epitopes among multi-variants. Pseudovirion-based neutralization results revealed that the antibody cocktail, 1D1 and 3D2, showed high potency in multiple variants of SARS-CoV-2 infection. *In vivo* studies showed the ability of the antibody cocktail treatment (intraperitoneal (*i*.*p*.) administration) to reduce viral load (Beta variant) in blood and various tissues. While the antibody cocktail treatment (intranasal (*i*.*n*.) administration) could not significantly reduce the viral load in nasal turbinate and lung tissue, it could reduce the viral load in blood, kidney, and brain tissue. These findings revealed that the efficacy of the antibody cocktail, 1D1 and 3D2, should be further studied in animal models in terms of timing of administration, optimal dose, and efficacy to mitigate inflammation in targeted tissue such as nasal turbinate and lung.

## Introduction

Coronavirus disease 2019 (COVID-19) is an infectious disease caused by severe acute respiratory syndrome coronavirus 2 (SARS-CoV-2) that has rapidly spread causing a worldwide pandemic [1–4]. The SARS-CoV-2 is an RNA virus with the characteristic of multiple spike glycoproteins on its envelope [5–10]. The receptor-binding domain (RBD) on the spike proteins binds specifically with the cellular receptor angiotensin-converting enzyme 2 (ACE2) of its host cells, resulting in a fusion cascade and virus entry [11–14]. This virus can transmit efficiently through respiratory droplets and aerosols with a reproduction number of up to 8 [15–17]. It replicates in the upper airway during the incubation period before developing symptoms. As of 13 September 2022, there have been 606,459,140 confirmed cases of COVID-19 globally, including 6,495,110 deaths reported to WHO [18].

Effective treatments such as monoclonal antibody therapy or antiviral drugs are available for alleviating symptoms and curing patients [19–25]. A monoclonal antibody with neutralizing activity against SARS-CoV-2 could potentially block the interaction between RBD/ACE2 to prevent virus entry and replication. Several spike protein-specific antibodies potentially have shown efficiency for neutralizing SARS-CoV-2 [26–32]. Some anti-SARS-CoV-2 monoclonal antibodies such as Sotrovimab, Casirivimab, Bebtelovimab, Imdevimab and Evusheld (tixagevimab co-packaged with cilgavimab) have already received emergency use authorizations from the Food and Drug Administration (FDA) for treatment of hospitalized COVID-19 patients [33–36].

Until now, the uncontrolled worldwide transmission has affected the evolutionary change of the virus due to genome mutation. A mutation might result in genetic variants of SARS-CoV-2 and could consequently impact SARS-CoV-2 properties such as transmissibility, immunity, and infection severity [37–40]. SARS-CoV-2 variants that meet the definitions of Variant of Interest (VOIs) and increase the transmissibility, virulence, and decrease in the effectiveness of treatments, vaccines, and diagnostics are called Variants of Concern (VOCs), which now includes Alpha, Beta, Gamma, and Delta [41,42]. Although previous studies showed several possible therapeutic mAbs, there is still a limited amount of anti-SARS-CoV-2 mAbs against other VOCs, which are currently the dominant variants since early 2021.

This study aimed to assess the newly developed highly potent mAbs which could be used against multiple variants of SARS-CoV-2 and provide therapeutic benefits to COVID-19 patients.

## Results

### Generation/Isolation of human monoclonal antibodies against RBD wild-type of SARS-CoV-2

**Fig 1:**
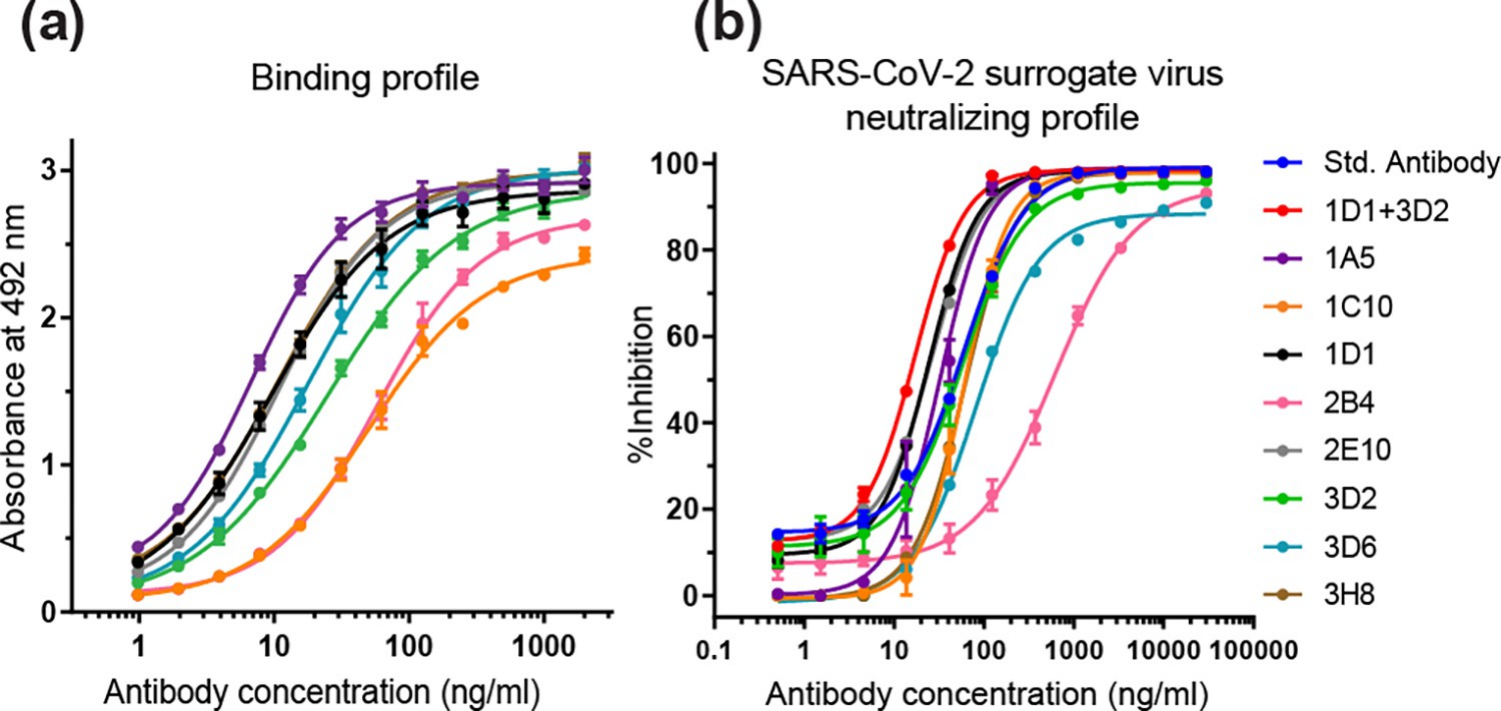
Binding and neutralizing profile of human antibodies against RBD protein (wild-type) of SARS-CoV-2. (a) ELISA binding profile of each antibody to RBD protein, and (b) cPass neutralizing profile of each antibody to RBD protein. Data are presented in mean ± SD (n = 3).

To develop a fully human monoclonal antibody against severe acute respiratory syndrome coronavirus 2 (SARS-CoV-2), human PBMCs were isolated from patients who had recovered from COVID-19. After generating human hybridoma cells using human hybridoma technology, these cells were cultured and screened for the clones that produce anti-RBD (wild-type) SARS-CoV-2. We were then able to successfully isolate the human hybridoma clones producing antibodies against RBD (wild-type) of SARS-CoV-2. The ELISA binding profile is shown in Fig 1A. Results demonstrated that all isolated antibodies exhibited high binding to the RBD (wild-type) of SARS-CoV-2. The 1A5 mAb displayed the highest binding activity. The EC_50_ values of binding activity of each antibody on RBD (wild type) are summarized in Table 1. The neutralization activity of isolated human antibodies on the SARS-CoV-2 surrogate (wild-type) infection was also evaluated by the cPass assay (Fig 1B). Results showed that the cocktail combination of 1D1 and 3D2 antibodies exhibited the highest neutralizing activity with the IC_50_ of 17.16 ng/ml. The IC_50_ values of each antibody on SARS-CoV-2 surrogate virus neutralizing activity are summarized in Table 1.

**Table 1:** ELISA binding activity, surrogate neutralizing activity, and binding kinetic data of human antibodies against RBD wild-type of SARS-CoV-2.

Antibodies	Binding activity, EC_50_ (ng/ml)	Surrogate neutralizing activity, IC_50_ (ng/ml)	Binding kinetics data				
			Antibody capture level (RU)	*k*_*on*_ (1/M.s)	*k*_*off*_ (1/s)	*K*_*D*_ (M)	t_1/2_ (min)
**Positive control**	N/A	60.21	N/A	N/A	N/A	N/A	N/A
**1D1 + 3D2**	N/A	17.16	N/A	N/A	N/A	N/A	N/A
**3D6**	17.64	68.42	68.58	1.12×10^6^	4.81×10^−5^	4.01×10^−11^	240.2
**3D2**	24.51	57.84	134.10	3.39×10^5^	2.68×10^−4^	7.89×10^−10^	43.1
**3H8**	10.73	52.27	159.70	3.05×10^5^	4.44×10^−5^	1.46×10^−10^	260.3
**1C10**	50.82	49.95	90.89	4.17×10^5^	6.36×10^−4^	1.53×10^−9^	18.2
**2B4**	57.85	612.30	65.95	9.52×10^5^	6.40×10^−4^	6.72×10^−10^	18.1
**1A5**	6.76	30.25	82.31	2.89×10^6^	1.27×10^−4^	4.39×10^−11^	91.0
**2E10**	10.97	26.82	104.00	7.20×10^5^	8.01×10^−5^	1.11×10^−10^	144.2
**1D1**	9.55	24.37	176.50	6.20×10^5^	1.50×10^−4^	2.42×10^−10^	77.1

N/A = Not available/ Not determined.

To determine the binding kinetics of human antibodies and RBD (wild-type) of the SARS-CoV-2 spike protein, the surface plasmon resonance (SPR) technique was performed by Biacore T200 equipped with a protein G sensor chip (GE Healthcare). Single-cycle kinetics at 25°C was used to determine the binding kinetics of each antibody and its target. The binding kinetic values are summarized in Table 1. All antibodies exhibited the equilibrium constant (*K*_*D*_) values in the subnanomolar range. The 3D6 antibody showed the highest binding affinity, with a *K*_*D*_ value of 4.01×10^−11^ nM, followed by 1A5, 2E10, 3H8, 1D1, 2B4, 3D2, and 1C10, respectively.

To evaluate the binding epitope of each human antibody to RBD wild-type of SARS-CoV-2 spike protein, the biolayer interferometry (BLI) technique was used to cluster these isolated antibodies. The experiment was performed using the ForteBio Octet HTX instrument using an in-tandem format. A biosensor was immobilized with a wild-type SARS-CoV-2 RBD-His tagged protein and then soaked with a saturating primary monoclonal antibody (1st mAb) followed by a competing secondary monoclonal antibody (2^nd^ mAb) in a pairwise combinatorial manner. The binding response was evaluated at each step after subtracting the self-blocking background signal. The Octet Data Analysis HT 10.0 software was used for data analysis with the Epitope Binning mode.

Epitope binning data of human antibodies are presented in Table 2. The results demonstrated that pairs of non-competing monoclonal antibodies could potentially be combined to form antibody cocktails (e.g., 3D2 and 1D1).

**Table 2:** Epitope binning data of human antibodies against RBD wild-type of SARS-CoV-2.

Step I prototype RBD-His capture (nm)	Step II measure 1^st^ mAb bound		Step III Measure 2^nd^ mAb bound (nm)							
	1^st^ mAb	1^st^ mAb bound (nm)	3D6	3D2	3H8	1C10	2B4	1A5	2E10	1D1
0.84±0.05	**3D6**	1.41 ± 0.04	0.00	1.08	1.03	1.03	0.85	1.02	1.12	1.13
	**3D2**	1.24 ± 0.02	0.92	0.00	0.89	0.82	0.63	0.86	0.87	0.84
	**3H8**	1.36 ± 0.03	1.10	1.08	0.00	-0.03	-0.04	0.02	-0.01	1.09
	**1C10**	1.23 ± 0.02	1.03	0.92	0.02	0.00	-0.05	0.04	0.01	0.05
	**2B4**	1.12 ± 0.01	1.07	1.04	0.10	0.02	0.00	0.15	0.06	0.13
	**1A5**	1.44 ± 0.01	1.16	1.10	-0.03	-0.07	-0.05	0.00	-0.06	0.00
	**2E10**	1.20 ± 0.04	0.83	0.73	0.04	0.00	-0.09	0.06	0.00	0.05
	**1D1**	1.29 ± 0.04	0.94	0.79	0.82	-0.04	-0.11	-0.01	-0.05	0.00

Grey = bi-directional competition, Light grey = no competition, White = self-competition.

### Neutralizing activity of highly potent human antibodies against multiple variants of SARS-CoV-2

To assess the neutralization activity of isolated human antibodies on multiple variants of SARS-CoV-2, the pseudovirus neutralization assay was performed. The pseudovirus particles comprise the S protein of SARS-CoV-2 expressed on the surface. These pseudoviruses can bind to the ACE2 overexpressed target cells and stimulate the fusion cascade of virus-target cells. After pseudovirus infection, infected-target cells will express the luciferase. By measuring the bioluminescence signal, the extent of pseudovirus infection can be determined. If the antibodies can neutralize the RBD, the interaction of ACE2 and S protein on the pseudovirus is blocked, resulting in a decrease in the bioluminescence signal.

**Fig 2:**
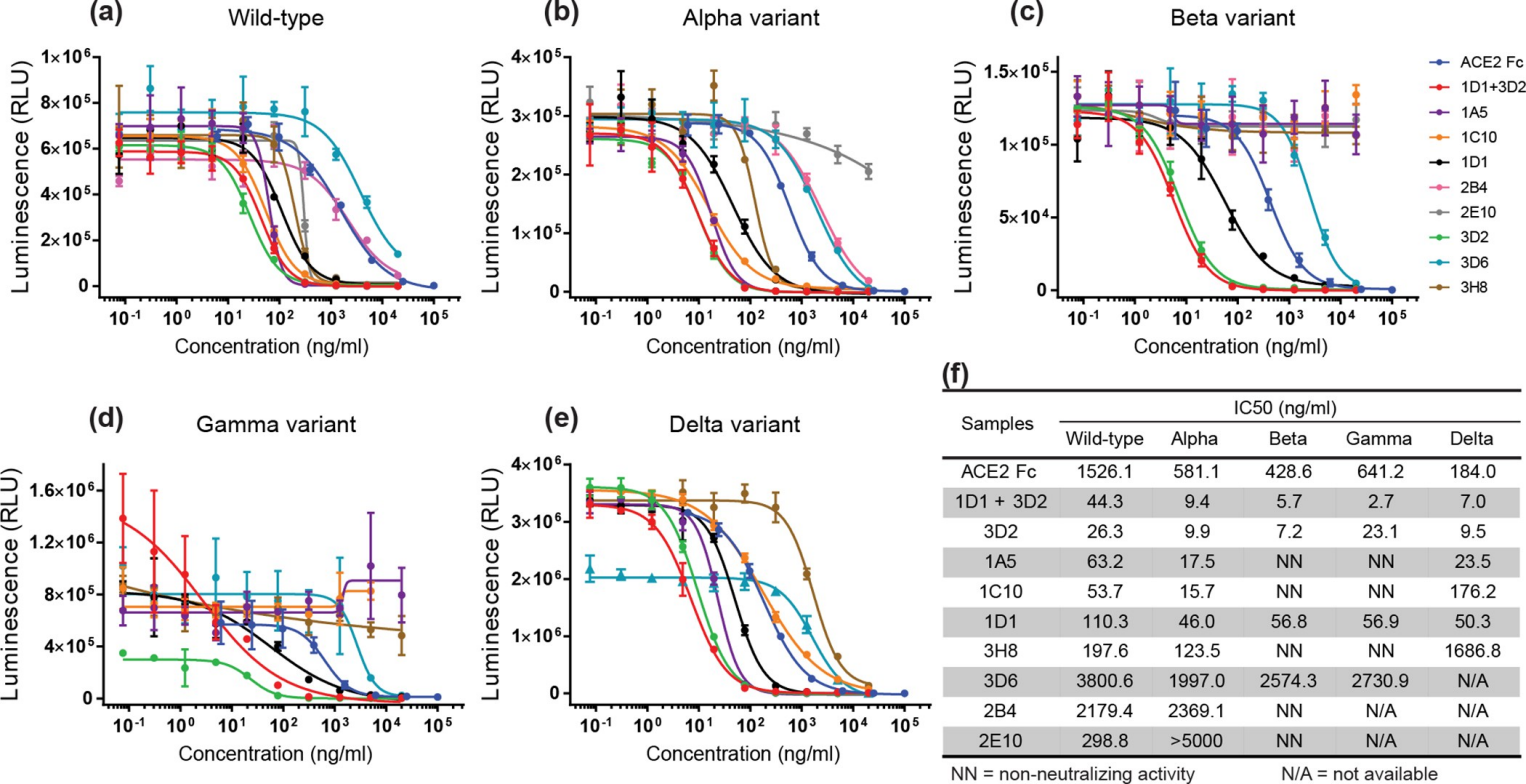
Pseudovirus neutralization activity of human antibodies against SARS-CoV-2 VOCs. Neutralizing profile of antibodies against (**a**) wild-type, (**b**) Alpha variant, (**c**) Beta variant, (**d**) Gamma variant, and (**e**) Delta variant of SARS-CoV-2. Data are presented in mean ± SD (n = 3). (**f**) The IC_50_ values of each human antibody on SARS-CoV-2 pseudovirus neutralization activity.

The pseudovirus neutralizing profiles of each antibody against multiple variants are demonstrated in Fig 2. The neutralization profiles of each human anti-SARS-CoV-2 antibody and antibody cocktail are depicted in Fig 2A–2E (wild-type, Alpha variant, Beta variant, Gamma variant, and Delta variant, respectively). The IC_50_ values of each antibody are represented in Fig 2F. The antibody cocktail, as well as 3D2 and 1D1 antibodies, demonstrated an IC_50_ value of <150 ng/ml for all SARS-CoV-2 variants. In addition, the antibody cocktail exhibited highly potent neutralizing activity against wild-type and the best potency in other VOCs of SARS-CoV-2.

### Authentic virus neutralizing activity of highly potent human antibodies against SARS-CoV-2 Delta variant

**Fig 3:**
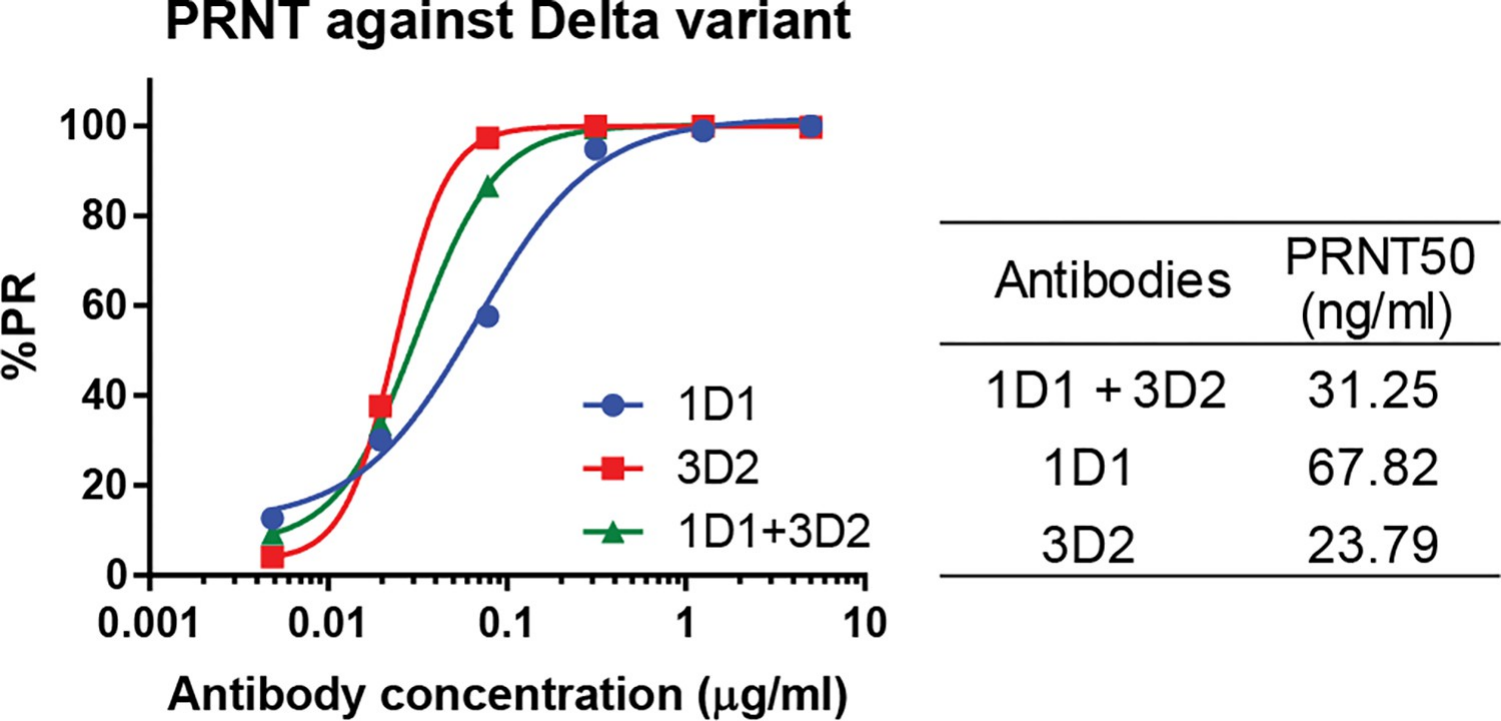
Neutralization efficacy of high-potency human antibodies against SARS-CoV-2 Delta variant assessed by the plaque reduction neutralization test (PRNT). Data are presented in mean ± SD.

One of the most crucial properties of antibodies is the ability to neutralize an authentic virus. Plaque neutralization assay using a live virus and Vero cells were used to evaluate neutralizing ability. The plaque-forming units of SARS-CoV-2 were counted by specific dyes that reacted with the infected cell. A specific antibody that can neutralize the virus will prevent the virus from forming plaque in the cell monolayer. The neutralization efficacy profile of antibodies against the SARS-CoV-2 Delta variant are displayed in Fig 3. When 1D1, 3D2, and antibody cocktail were mixed with the virus and then plated onto cells, it was found that the concentration of plaques was reduced with PRNT_50_ to less than 70 ng/ml.

### X-ray crystallography structure of the complex between high-potency human antibodies and RBD of SARS-CoV-2

**Fig 4:**
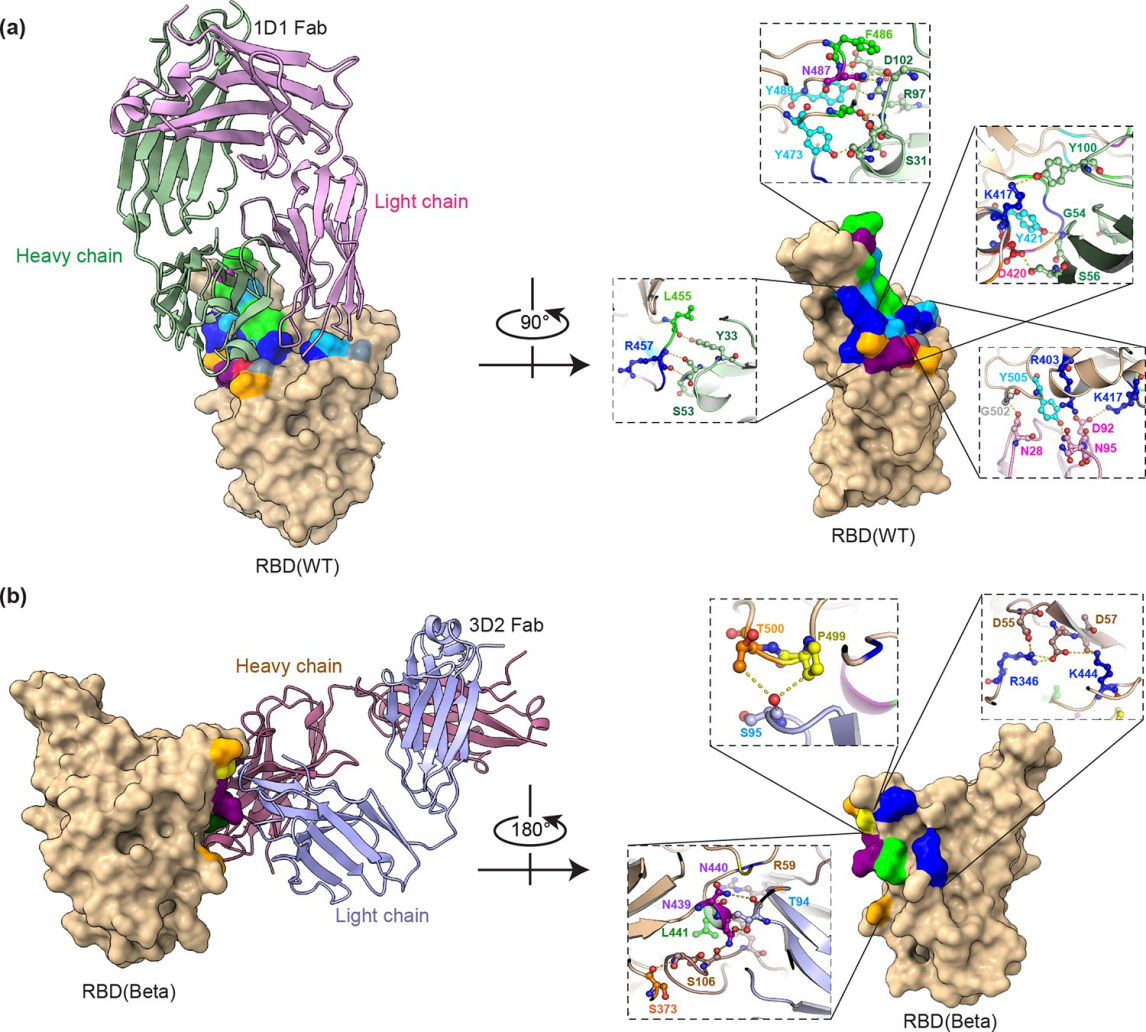
X-ray crystallography structures of two Fab fragment complexes. (a) 1D1 Fab complexed with the wild-type RBD protein, and (b) 3D2 Fab complexed with the Beta RBD protein. The amino acids in the binding epitopes are displayed using the CINEMA color scheme, with Asp and Glu represented in red, His, Lys, and Arg represented in blue, Ser and Thr represented in orange, Asn and Gln represented in purple, Phe and Tyr represented in cyan, Ala, Val, Leu, Ile, and Met represented in green, Pro represented in yellow, and Gly represented in gray. The zoom-in images show the contact interfaces between the Fab fragments and RBD proteins at atomic resolution.

In order to determine the binding epitope of high-potency antibodies to RBD spike proteins, X-ray crystallography was performed. The Fab fragments were prepared from the purified antibodies using the FabALACTICA Fab Kit (Genovis). Two Fab RBD complexes were produced, the 1D1 Fab formed a complex with the RBD (wild-type) and the 3D2 Fab formed a complex with the RBD (Beta variant) of SARS-CoV-2. To investigate the interactions between antibodies and the RBD protein, the crystals structures of the two Fab RBD complexes were determined (Fig 4). The 1D1 Fab RBD (wild-type) complex at 1.9 Å resolution and the 3D2 Fab RBD (Beta variant) complex at 2.2 Å. The result showed that the 1D1 Fab and 3D2 Fab bind to different epitopes on RBD proteins, i.e. 1D1 Fab and 3D2 Fab bind to class I&II and class III RBD binding epitopes, respectively [43]. The lists of binding residues between the Fabs and RBD proteins are shown in S2 and S3 Tables.

### *In vivo* studies of high-potency human antibodies against SARS-CoV-2 administered *i*.*p.*

**Fig 5:**
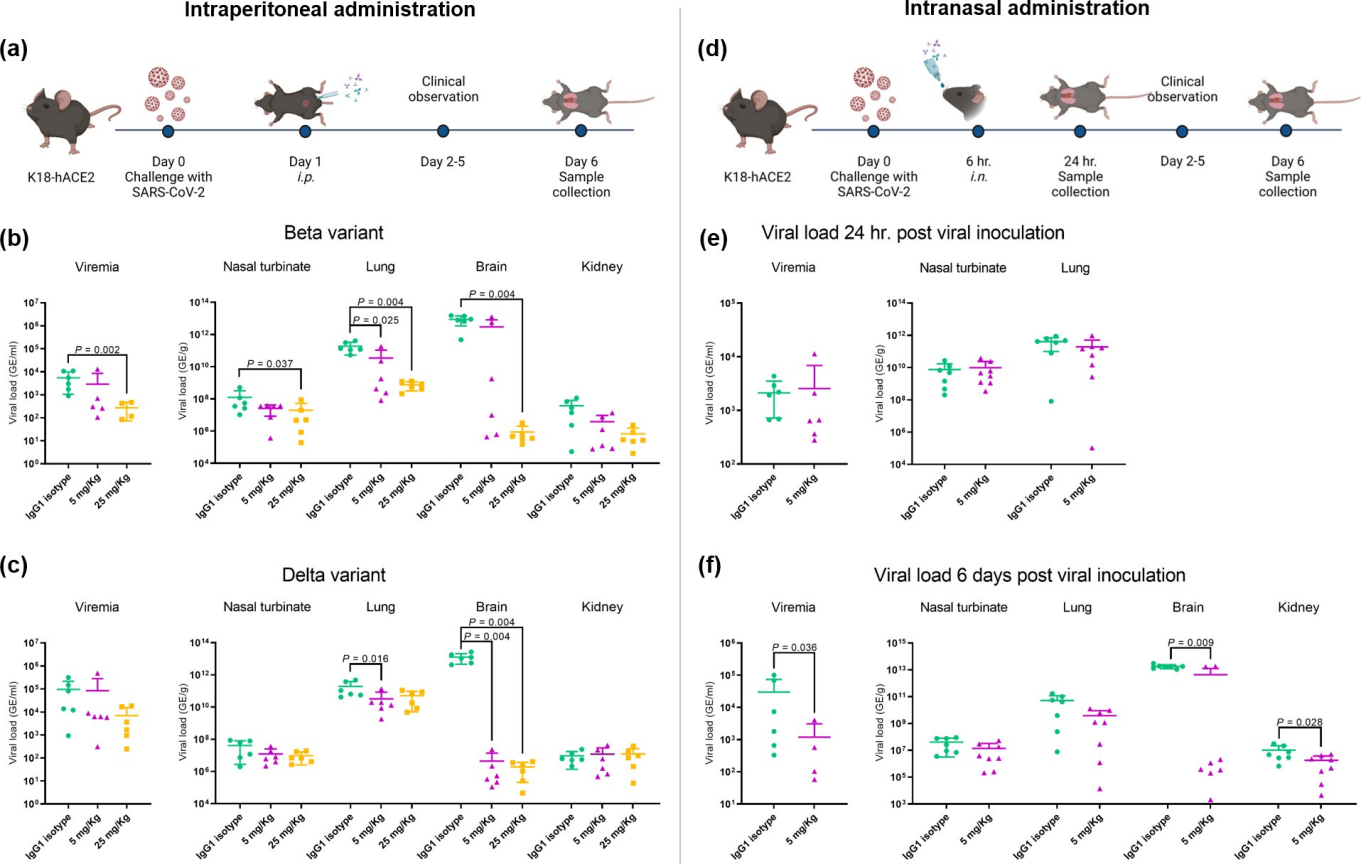
*In vivo* therapeutic efficacy of high-potency human antibodies on SARS-CoV-2 infection. (**a**) Experimental design of the intraperitoneal (*i*.*p*.) administration therapy. For the *i*.*p*., mice were challenged with SARS-CoV-2 either Beta variant or Delta variant (n = 6 per variant). The antibody cocktail at 5 mg/Kg (medium dose) and 25 mg/Kg (high dose) were used as treatments, while the human IgG1 isotype antibody was used as a negative control (25 mg/Kg) (created with Biorender.com). Viral titers in different organs were evaluated 6 days post-challenge with (**b**) Beta variant and (**c**) Delta variant of SARS-CoV-2. (**d**) Experimental design of the intranasal administration (*i*.*n*.) therapy. For the *i*.*n*., mice were challenged with SARS-CoV-2 Delta variant and treated with either the antibody cocktail (5 mg/Kg) or human IgG1 isotype antibody (5 mg/Kg) 6 hours post-viral inoculation (created with Biorender.com). Viral titers in different organs were evaluated at (**e**) 24 hours and (**f**) 6 days post-viral inoculation. Data are presented in mean ± SD. A *P-value* of less than 0.05 was considered statistically significant.

To demonstrate antiviral potency of the antibody, the IgG1 isotype control and the antibody cocktail were administered systemically (*i*.*p*.) 24 hours after inoculation with either the Beta or Delta variant of SARS-CoV-2 (Fig 5A). Subsequent reductions in viremia and viral distribution in tissues from all four major organs were evaluated by qRT-PCR. The clinical presentations of mice in all treatment groups were unremarkable for 4 days following viral inoculation. For all groups, reduced appetite was the only abnormal clinical observation noted during this period. This observation was typically seen in less than 50% of the mice. However, on Day 5 of the experiment, mice began to exhibit notable clinical symptoms of disease. In particular, IgG1 isotype-treated mice infected with either the Delta or Beta variant began reaching early-euthanasia criteria. By the end of the experiment on Day 6, the severity of the clinical symptoms of disease in all IgG1 isotype-treated mice met the threshold for early euthanasia. For groups infected with the Beta variant, however, both the high and medium dose mAb treatment appeared to provide substantial prevention of clinical disease. At high dose levels, no mice reached the threshold for early euthanasia, and no mice ever received a clinical score greater than one. For mice that received medium dose mAb treatment, only one mouse exhibited clinical symptoms severe enough to meet criteria for early euthanasia.

Regarding expression of viral RNA within Beta variant infected groups (Fig 5B), qRT-PCR found detectable levels of viral RNA in all groups. However, the high dose antibody cocktail treatment (2.71×10^2^ GE/ml) significantly reduced the levels of viremia as compared to the IgG1 isotype -treatment group (5.44×10^3^ GE/ml). The medium dose antibody cocktail treatment (2.89×10^3^ GE/ml) also resulted in a numerical reduction in viremia compared to the IgG1isotype-treatment group. Substantial viral RNA was detectable in all four major organs examined in all animals. In the IgG1 isotype-group, the highest level of mean viral RNA was detected in the brain (8.82×10^12^ GE/g) followed by lung (1.92×10^11^ GE/g), nasal turbinate (1.26×10^8^ GE/g) and kidney (3.76×10^7^ GE/g). The cocktail treatment reduced viral RNA in a dose dependent manner for all tissues when compared to the IgG1 isotype-treated group. More specifically, the high dose mAb treatment significantly reduced viral RNA in nasal turbinate (*p*-value = 0.037), brain (*p*-value = 0.004) and lung tissues (*p*-value = 0.004), whereas the medium dose mAb treatment significantly reduced the level of viral RNA in lung tissue only (*p*-value = 0.025).

For groups infected with the Delta variant (Fig 5C), viral RNA was detected in 100% of mice in each treatment group. Furthermore, while both the high dose mAb treatment (6.94×10^3^ GE/ml) and the medium dose mAb treatment (8.47×10^4^ GE/ml) resulted in a numerical reduction in viremia compared to IgG1 isotype-treatment (9.50×10^4^ GE/ml), neither reduction reached statistical significance. Reduction of viral RNA in brain (*p*-value = 0.004) and brain/lung (*p*-value = 0.004/0.016) tissues was found in the high and medium dose mAb treatment groups, respectively. However, between the high and medium dose groups, no significant differences in viral RNA were found in any of the tissues.

### *In vivo* studies of high-potency human antibodies against SARS-CoV-2 administered *i*.*n.*

Following the demonstration of antiviral effects of *i*.*p*. administration of the mAb cocktail, a second cohort of SARS-CoV-2 infected animals was leveraged to test for efficacy following *i*.*n*. administration (Fig 5D). IgG1 isotype-treatment and antibody cocktail (5 mg/Kg) treatments were administered *i*.*n*. 6 hours post inoculation with the Delta variant. Similar to *i*.*p*. administration, administration of mAbs *i*.*n*. resulted in an improvement in clinical symptoms and mortality rates (71.4% mortality in IgG1 isotype treated animals compared to 25% mortality in mAb treated animals). Moreover, a statistically significant decrease in body weight of IgG1 isotype treated mice was found compared to those treated with the mAb cocktail. Reduction of viremia and viral distribution in tissues were also evaluated by qRT-PCR at 1-day and 6-days post infection. At 1-day post infection (Fig 5E), there was no significant difference of viremia and viral RNA levels in nasal turbinate and lung tissues between IgG1 and antibody cocktail treatment groups. However, IgG1 treated mice showed detectable viremia with a mean of 2.12×10^3^ GE/ml whereas antibody cocktail treated mice showed detectable viremia with a mean of 2.55×10^3^ GE/ml. Comparable levels of viral RNA were expressed in nasal turbinate between groups (7.59×10^9^ and 9.91×10^9^ GE/g for IgG1 and mAb treatment groups, respectively). A numerical reduction in viral RNA levels was found in the lung tissue of the mAb-treated group (1.93×10^11^ GE/g) compared to the IgG1-treated group (4.08 × 10^11^ GE/g), however, this reduction failed to reach statistical significance.

The 6-day post infection results are displayed in Fig 5F. Antibody cocktail treatment significantly reduced viremia compared to IgG1 treatment with only antibody cocktail-treated mice exhibiting detectable viremia compared to IgG1-treated mice exhibiting detectable viremia. For viral RNA in tissue samples, no significant differences were found in nasal turbinate tissue (4.13×10^7^ and 1.39×10^7^ GE/g for IgG1 and mAb treated mice, respectively) and lung tissues (5.37×10^10^ and 3.88×10^9^ GE/g for IgG1 and mAb treated mice, respectively). However, mAb treatment significantly reduced the levels of viral RNA in the brain and kidneys.

## Discussion

In our present study, we successfully generated human monoclonal antibodies using hybridoma technology and successfully isolated the human hybridoma clones producing antibodies against the RBD protein (wild-type) of SARS-CoV-2. We characterized mAbs that were isolated from B cells of recovered COVID-19 patients. The isolated mAbs showed sufficient specificity and sensitivity to bind and neutralize the RBD protein (wild-type) of SARS-CoV-2 assessed by ELISA and cPass assay respectively. For the binding kinetics assay, data showed that all antibodies exhibited the equilibrium constant (*K*_*D*_) values in the subnanomolar range. Interestingly, two of these isolated mAbs, 1D1 and 3D2 targeted non-competing epitopes on RBD. 1D1 and 3D2 antibodies were combined to form mAb cocktail to develop a product that had an improved chance of maintaining efficacy against future variants of SARS-CoV-2. These antibodies showed high pseudo-virus neutralization of Wild-type, Alpha, Beta, Gamma, and Delta VOCs. In addition, the PRNT results also confirmed that this pair of the mAbs had high potency in the protection of SARS-CoV-2 Delta variant infection. The crystal structures of highly potent neutralizing antibodies (1D1 and 3D2 monoclonal antibodies) were also obtained to determine the binding area between these antibodies and the RBD proteins of SARS-CoV-2. Data exhibited that the 1D1 and 3D2 antibodies binded to the RBD protein at different epitopes. When defining the epitope of our antibodies with the epitope landscape of the RBD protein, it was found that 1D1 was grouped in RBD class 2 binding antibody. While 3D2 was classified as an RBD class 5 binding antibody, a conserved region of RBD, resulting in 3D2 providing neutralizing activity against all variants [44].

To evaluate *in vivo* activity, we assessed the therapeutic efficacy of our mAb cocktail using an established model of SARS-CoV-2 infection in hACE2-expressing K18 transgenic mice. Intraperitoneal (*i*.*p*.) administration was specifically designed as the first assessment. Mice were infected with either SARS-CoV-2 Beta or Delta variant one day before mAb cocktail injection. Following *i*.*p*. administration, the mAb cocktail was able to dramatically decrease the morbidity and mortality of mice infected with the Beta variant of SARS-CoV-2 compared with the IgG1 isotype control. These findings were mirrored by the molecular findings that showed significant reductions in viral replication in the blood and lungs. Taken together, these data suggested that the mAb treatment could not only reduce viral load in the blood and lungs, but that this decrease translated into clinically relevant outcomes (i.e., decreased morbidity and mortality among treated subjects). Interestingly, the improvement in clinical symptoms following *i*.*p*. administration was not as dramatic in the Delta variant infected mice compared to mice infected with the Beta variant. This could be an indication that effective dosing regimens may vary depending on the variant of virus in question. Given that reductions in clinical symptoms and viral replication were still demonstrated against the Delta variant, it was likely that the mAbs do exert a cross-variant antiviral effect. Future studies may need to consider adjusting dose, route of administration, or frequency of dosing to determine the most efficacious treatment regimens for a given SARS-CoV-2 variant.

Intranasal (*i*.*n*.) administration was tested as a follow up administration route which is more clinically relevant. The delta infected mice received either mAb cocktail treatment or the IgG1 isotype control. Similar to the *i*.*p*. administration study, evaluation of the protective effects of *i*.*n*. administration of mAbs demonstrated antiviral efficacy. Notably, there were significant improvements in morbidity and mortality of Delta infected mice that received mAb treatment compared to mice that received the IgG1 isotype control. This improvement was supported by statistically significant reductions of circulating viral loads in the blood, and numerical decreases of viral load in the respiratory tract. This again suggests that *i*.*n*. mAb therapy has the capability of reducing SARS-CoV-2 viral replication to an extent that can translate into relevant positive clinical outcomes. It should be noted that while viral loads in the respiratory tract were reduced, these decreases failed to reach statistical significance. This is another indication that modifications of the dosing regimen still may need to be addressed in future studies to ensure that optimal regimens are identified for future clinical implementation.

The WHO has designated Omicron as a SARS-CoV-2 VOC, which became dominant in many countries during early 2022. Our study was limited by the fact that our testing stages started in 2020–2021 before Omicron was prevalent [45–48]. The Omicron variant contains more than 30 mutations on the virus’s spike protein [46,49], resulting in rapid spread and large outbreaks in 2022. However, Omicron is less severe than previous variants [50], according to the CDC.

In conclusion, this study demonstrated that this novel mAb cocktail exerts protective effects against two variants of SARS-CoV-2. Moreover, this protective effect was demonstrated in two different routes of administration and dosing regimens. The results of this study illustrate the potential of this mAb cocktail as a viable multi-variant intervention against SARS-CoV-2 infection. However, future studies need to consider adjusting dose, route of administration, frequency of dosing, testing with new/different strains and pathology for future clinical implementation. Moreover, for using the mAb cocktail as a nasal spray solution as a medical device, future studies may need to evaluate the preclinical studies following the ISO 10993 standards with good biocompatibility based on cytotoxicity, skin sensitization, and intracutaneous reactivity as well as satisfactory safety profiles by acute and subacute systemic toxicity before starting clinical trials.

## Materials and methods

### Ethical statement

This project was approved by the Institutional Review Board on Human Research of the Faculty of Medicine, Chulalongkorn University with certificate of approval number 814/2020. Written informed consent was obtained from all subjects ≥ 18 years old who were informed of the risks and signed a consent form before enrolling in the study. The study was conducted according to the Helsinki Declaration and Good Clinical Practice guidelines. All experimental procedures involving animals were conducted under a protocol approved by the Institutional Animal Care and Use Committee of AFRIMS. This animal protocol was executed in compliance with Thai laws, the Guide for the Care and Use of Laboratory Animals, the Animals for Scientific Purposes Act (National Research Council of Thailand, 2015), the Animal Welfare Act, and all applicable U.S. Department of Agriculture, Office of Laboratory Animal Welfare, and U.S. Department of Defence guidelines.

### Isolation of PBMCs from recovered COVID-19 patients

Blood was collected from 10 hospitalized and recovered COVID-19 patients with high IgG titers against the SARS-CoV-2 spike protein from June to July 2020. The blood samples of approximately 5 ml were allowed to clot at room temperature for 15 minutes, and then centrifuged at 2000×*g* for 10 minutes to isolate the serum. The serum was aliquoted and subsequently used for measuring the level of neutralizing antibodies against wild-type SARS-CoV-2 by plaque-reduction neutralization test (PRNT). PBMCs from the four patients with the highest PRNT activities were isolated by the Ficoll™ density gradient centrifugation technique. B cells were isolated from PBMCs by EasySep™ human B cell isolation kit (STEMCELL Technologies) following the manufacturer’s instructions.

### Generation of human antibodies against SARS-CoV-2

To generate human antibodies using hybridoma technology, isolated B cells from recovered COVID-19 patients were transformed by Epstein-Barr virus (EBV) obtained from the B95-8 lymphoblastoid cell line supernatant. The EBV-transformed B cells were cultured in 384-well plates at 37°C for seven days. Cells in each well were then transferred to 96-well plates containing irradiated heterologous human PBMC feeder cells. After incubation for three days, the supernatant from each well was screened based on binding and neutralizing activities against wild-type SARS-CoV-2 by ELISA. Next, EBV-transformed B cells with the top-ranked neutralizing activities were fused with SHM-33 myeloma cells using the cell electrofusion technique to create stable human hybridoma clones. Briefly, individually selected EBV-transformed B cell pools were mixed with myeloma cells followed by washing with BTX cytofusion medium. The cell mixture was transferred to a cytofusion cuvette, and electrofusion was performed using BTX ECM 2001 fusion system. After fusion, hybridoma cells were cultured in 96-well plates for ten days in a ClonaCell-HY Medium E (STEMCELL Technologies) containing hypoxanthine-aminopterin-thymidine (HAT). The supernatant from each well was screened based on binding and neutralizing activities against wild-type SARS-CoV-2 by ELISA.

Hybridoma cells with high neutralizing activities were subcloned by a limiting dilution technique to achieve hybridoma monoclonality. After 7–10 days of culturing, hybridoma clones were screened by ELISA. Finally, selected human hybridoma clones with high neutralizing activities were expanded to produce SARS-CoV-2 neutralizing antibodies.

### Screening of human antibodies against SARS-CoV-2 by binding activity

ELISA technique was utilized to screen for high binding anti-SARS-CoV-2 antibodies. Each well of the MaxiSorp 96-well ELISA plate (ThermoFisher Scientific) was coated with 10 ng of wild-type SARS-CoV-2 RBD-His proteins (Sino Biological) at 4°C overnight. The coated ELISA plate was washed and blocked with 0.05% Tween-20 in the PBS buffer (PBST). Next, 100 μl culture supernatant samples from EBV-transformed B cells or human hybridoma cells were added to the coated wells. The plate was incubated for 1 hour at 37°C and washed three times with PBST. A secondary antibody probing step was performed by adding 100 μl/well of goat anti-human IgG (Fcγ fragment specific)-HRP (Jackson ImmunoResearch), incubating for 1 hour at 37°C and washing with PBST 3 times. Next, the plate was added with o-Phenylenediamine dihydrochloride (OPD) substrate solution (100 μl/well) and incubated in the dark for 20 minutes at room temperature. The reaction was stopped by adding 2N H_2_SO_4_ solution (50 μl/well). Finally, the absorbance was measured at 492 nm by Cytation 5 (BioTek).

### Screening of human antibodies against SARS-CoV-2 by neutralizing activity

ELISA technique was employed to evaluate the RBD/ACE2 neutralizing activity from EBV-transformed B cells and human hybridoma cells that produced RBD-specific antibodies (positive binding clones). First, an ELISA plate was coated with recombinant ACE2 human Fc tag proteins (Genscript) at 4°C overnight. The coated ELISA plate was washed and blocked with PBST. Next, 50 μl culture supernatant samples from the positive binding clones or a control antibody were pre-incubated with 50 μl of RBD mouse Fc tag proteins (Genscript) at 37°C for 30 minutes and individual samples were transferred to the coated plate. After incubation at 37°C for 1 hour, the plate was washed by PBST three times. Goat anti-mouse Ig (γ-chain specific)-HRP (Jackson ImmunoResearch) was added to the reaction, then incubated at 37°C for 1 hour to detect RBD mouse Fc tag proteins. The plate was washed three times by PBST before adding 100 μl of OPD and then incubated in the dark at room temperature for 20 minutes. The stop solution was added to each well, and the absorbance at 492 nm was determined by Cytation 5 (BioTek).

### Sequencing of human antibody variable regions

Sequencing was performed by whole transcriptome shotgun sequencing technique (RNA-Seq). In brief, total RNA was extracted from Human hybridoma clones producing neutralizing antibodies against SARS-CoV-2 disclosed herein. A barcoded cDNA library was generated through RT-PCR using a random hexamer. Next-generation sequencing was performed on an Illumina HiSeq sequencer. Contigs were assembled, and data was mined for all viable antibody sequences (i.e., those not containing stop codons). Sequence analysis was performed separately to identify variable heavy and variable light domains. The complementarity determining regions (CDRs) were identified using the Kabat definition.

### Binding profile of human monoclonal antibodies against SARS-CoV-2

The Expi293 transient expression system (Gibco™) was used to produce recombinant human monoclonal antibodies against SARS-CoV-2. The binding profile of purified antibodies disclosed herein was assessed by ELISA, as mentioned above (Screening of human antibodies against SARS-CoV-2 by binding activity) with the following modification. Instead of using the culture supernatant, purified antibodies at 2 μg/ml were used as starting samples, then 11 rounds of 2-fold dilution were performed for each sample until the final concentration of 0.977 ng/ml was reached.

### Binding kinetics of human monoclonal antibodies against SARS-CoV-2

To determine the binding kinetics of human monoclonal antibodies disclosed herein and RBD of wild-type SARS-CoV-2 spike protein, the surface plasmon resonance (SPR) technique was performed by Biacore T200 equipped with a protein G sensor chip (GE Healthcare). For the antibody-capturing step, each purified human anti-SARS-CoV-2 monoclonal antibody (at 1 μg/ml prepared in the HBS-EP buffer) was injected into an individual flow cell in the sensor chip. Single-cycle kinetics at 25°C was determined by sequentially injecting recombinant RBD-His tag proteins at different concentrations. An HBS-EP buffer blank was also included as a negative control for baseline subtraction. The antibody binding affinity (*K*_*D*_) value was calculated using the Biacore T200 Evaluation Software v3.1.

### SARS-CoV-2 surrogate virus neutralization test of human monoclonal antibodies

The SARS-CoV-2 surrogate virus neutralization test (cPass, GenScript) was used to assess the neutralizing activity of antibodies disclosed herein. Briefly, the individual human anti-SARS-CoV-2 mAbs or antibody cocktail (1D1 + 3D2 in ratio 1:1 w/w) was mixed with wild-type RBD-HRP tag proteins and incubated at 37°C for 30 minutes. The control antibody (GenScript, A02087) that can neutralize spike proteins was used as a positive control. The mixture was then added to each well of the human ACE2-coated plate. After incubation at 37°C for 15 minutes, the plate was washed 4 times with a washing solution. In consequence, the free and non-neutralizing antibody-bound HRP-RBD were captured on the plate. Next, the plate was added with 100 μl TMB substrate solution to each well and incubated in the dark for 20 minutes. Finally, a stop solution was subsequently added to each well. The absorbance of the final solution was determined at 450 nm immediately.

### Epitope binning assay of human monoclonal antibodies against RBD wild-type protein of SARS-CoV-2

Biolayer interferometry (BLI) technique was used to cluster recombinant human monoclonal antibodies disclosed herein based on binding epitopes on RBD of wild-type SARS-CoV-2 spike protein. The experiment was performed using ForteBio Octet HTX in an in-tandem format, i.e., a biosensor was immobilized with a wild-type SARS-CoV-2 RBD-His protein and then presented with a saturating monoclonal antibody (1^st^ mAb) followed by a competing monoclonal antibody (2^nd^ mAb) in a pairwise combinatorial manner. The assays were performed at 32°C with a shaking speed of 1,000 rpm. Briefly, a wild-type SARS-CoV-2 RBD-His protein (3 μg/ml solution) was allowed to be captured onto an anti-His (AHC) biosensor tip for 300 seconds. The first and second anti-SARS-CoV-2 monoclonal antibodies (10 and 5 μg/ml, respectively) were sequentially presented to the biosensor tip for 300 seconds. The binding response was evaluated at each step after subtracting the self-blocking background signal. The data analysis was performed with Octet Data Analysis HT 10.0 software using the Epitope Binning mode.

### SARS-CoV-2 pseudovirus neutralization assay of human monoclonal antibodies

Pseudovirus Neutralization Assay kit (Luc reporter, GenScript) was used to evaluate the potency of human anti-SARS-CoV-2 mAbs and antibody cocktail for neutralizing infectivity of SARS-CoV-2 (Wild-type, Alpha (B.1.1.7), Beta (B.1.351), Gamma (P.1), and Delta (B.1.617.2) variants). In brief, the mAbs were diluted in 4-fold serial dilution with Opti-MEM, and 25 μl of the solution was transferred to an assay plate. Then, 25 μl of pseudovirus with luciferase reporter in Opti-MEM was added to wells and incubated at room temperature for 1 hour. Next, 50 μl of Opti-HEK293/ACE2 single-cell suspension at 600,000 cells/ml was added into each well. After 24 hours of incubation at 37°C, 50 μl of fresh media was added to the assay plate and incubated at 37°C for 24 hours. The culture supernatant was then carefully discarded using a pipette. Finally, luciferase reagent (50 μl) was added to the assay plate and subsequently incubated for 5 minutes at room temperature, and the bioluminescence signal was measured.

### Plaque reduction neutralization test (PRNT) of human monoclonal antibodies

Plaque reduction neutralization assay was used to measure the neutralization activity of mAbs and antibody cocktail against the SARS-CoV-2 Delta variant. In brief, 6,000,000 cells of Vero cells were plated into a 6-well assay plate. The plate was incubated at 37°C for 24 hours. Then, antibodies were prepared by diluting in MEM media supplement with 2% FBS and mixed with a plaque of live-SARS-CoV-2 virus. This antibodies-virus solution was incubated at 37°C for an hour. After that, the culture supernatant of prepared Vero cells was discarded and replaced by 200 μl antibodies-virus solution and 2800 μl new culture media supplemented with 1% methylcellulose, 1% 10,000 units/ml Penicillin-10,000 μg/ml Streptomycin and 10% FBS. The place assay was incubated for 7 days in a 37°C atmosphere. Finally, the viruses were then stained and counted for a percentage of plaque reduction calculation.

### Crystallization and crystal structure determination of antibodies and RBD protein

To determine the binding epitope of antibody and RBD by crystallography, the antibodies and RBD were expressed in small-scale production using bacmid transformation into High Five™ insect cells. The large-scale production was carried out to reach a sufficient amount of antibodies for crystallization. Then, proteins were purified and concentrated to an appropriate concentration for crystallization. The Fab fragments were prepared from the purified antibodies using the FabALACTICA Fab Kit (Genovis). The RBD protein was mixed with each Fab fragment at a 1,1:1 molar ratio and purified on a HiLoad Superdex 200 16/60 pg gel filtration column (Cytiva/GE Healthcare) using 20 mM Tris-HCl pH 7.4, 150 mM NaCl as mobile phase. For crystallization of 1D1 Fab RBD a concentration of 9.4 mg/ml and the above buffer was used. The setup used was 100 nl + 100 nl sitting drops in MRC plates over a reservoir of 40 μl. The crystals used for data collection were grown in the BCS screen (Molecular Dimensions) over the reservoir: 18% PEG Smear Broad, 0.08 M MgCl_2_, 0.08 M tri-sodium citrate and 0.1 M Bis-Tris pH 6.0. Crystals were flash-frozen in liquid nitrogen after addition of cryo-solution containing the reservoir solution with the addition of 25% glycerol. Data were collected at 100 K at station BioMAX, MAX IV, Lund, Sweden (λ = 0.9537 Å). The beamline is equipped with an Eiger 16M hybrid-pixel detector. Data was processed with autoPROC [51] using XDS [52] and Aimless [53]. The structure was determined by molecular replacement using the Phaser software [54]. One 1D1 Fab RBD complex was found in the asymmetric unit in space group P2_1_2_1_2_1_. The structure was refined in Refmac5 [55] and model building was done in Coot [56].

For crystallization of 3D2 Fab RBD (Beta) a concentration of 10.7 mg/ml and the same buffer as above. Initial crystals were obtained from the JCSG+ screen (Molecular Dimensions) containing 0.04 M potassium dihydrogen phosphate, 16% w/v PEG 8000, 20% v/v glycerol. The initial crystals were used for seeding and crystal used for data collection were grown in a reservoir solution of 0.04 M potassium dihydrogen phosphate, 18% w/v PEG 8000, 20% v/v glycerol and were obtained using seeds. Crystals were flash-frozen in liquid nitrogen after addition of cryo-solution containing: 40 mM potassium dihydrogen phosphate, 16% w/v PEG 8000, 10 mM Tris pH 7.4 and 25 v/v % glycerol. Data were collected at 100 K at station BioMAX, MAX IV, Lund, Sweden (λ = 0.9762 Å). The beamline is equipped with an Eiger 16M hybrid-pixel detector. Data was processed with XDSAPP [57] using XDS [52] and Aimless [53]. The structure was determined by molecular replacement using the Phaser software [54]. One RBD_B1.351:3D2 Fab complex was found in the asymmetric unit in space group C2. Refinement was done in Refmac5 [55] and model building was done in Coot [56]. For final data and refinement statistics, see the S1 Table.

### *In vivo* assessment of anti-viral effects of antibody cocktails against SARS-CoV-2 infection

#### Animals

Sixty-six [66] K18-hACE2 transgenic mice (female, 8–12 weeks of age, 17–25 grams) expressing human ACE2 were acquired from Jackson Laboratory USA. Mice were housed in an AALAC accredited vivarium at the Armed Forces Research Institute of Medical Sciences (AFRIMS) with ad libitum access to food, water, and environmental enrichment. Animals were randomly assigned (using Microsoft Excel random number generator function) to one of two cohorts (n = 36 for Cohort 1, n = 30 in Cohort 2) to assess the effects of the mAb cocktail following two different routes of administration (i.e., intraperitoneal (*i*.*p*.) and intranasal (*i*.*n*.)). All mice were group housed prior to the start of experimentation. For all *in vivo* experiments, animals received regular daily health checks by trained veterinary staff to assess pain and distress. Animals experiencing pain/distress were selected for early euthanasia. The method of euthanasia used for all animals (both those euthanized early for humane reasons as well as those that reach the end of the study) was CO_2_ inhalation. During euthanasia, animals were exposed to a CO_2_ flow rate ranging from 30% - 70% of cage volume/min until 1 minute after signs of respiration stopped. No exclusion criteria was set, and no animals (including those meeting early euthanasia criteria) were excluded from the study. To minimize potential confounders, animal treatments and assessments were performed in the order of cage number rather than by group assignment.

#### Viruses and viral intranasal challenge

The efficacy of the mAb cocktail was evaluated against the Beta and Delta variants of SARS-CoV-2. The Beta variant strain used was B.1.351 hCoV-19/South Africa/KRISP-EC-K005321/2020, and the Delta variant strain used was B.1.617.2 hCoV-19/Thailand/CU-A21287-NT/2021. All viruses were propagated in Vero E6 cells in a BSL-3 laboratory, and inocula of 2×10^4^ PFU/50 μl were prepared and stored until use during challenge. Immediately prior to the viral challenge, mice were moved to the AFRIMS BSL-3 vivarium. They were subsequently anesthetized with a combination of ketamine (40 mg/ml), xylazine (2 mg/ml), and atropine (0.06 mg/ml) at a dose of 0.1–0.3 ml per 100 grams of body weight administered intramuscularly. Once fully anesthetized, mice were placed in a supine position and 50 μl of inoculum was slowly administered dropwise into the nares to establish a respiratory infection. The mice were then immediately held upright for 30–60 seconds to allow inoculum to drain back into the sinuses. Once mice were fully recovered from anaesthesia, they were housed singly in the AFRIMS BSL-3 vivarium.

#### Intraperitoneal (*i*.*p*.) administration of mAb cocktail in mice infected with either the Beta or Delta variant of SARS-CoV-2

Mice from Cohort 1 (n = 36) were randomly assigned (using Microsoft Excel random number generator function) to one of two groups and were subsequently infected with either the Beta variant or Delta variant of SARS-CoV-2 as described above (n = 18 for the Beta infected group, n = 18 for the Delta-infected group). At 24-hours post inoculation, infected mice were divided into 3 subgroups (n = 12 per subgroup; 6 Beta-infected mice and 6 Delta-infected mice) to test the anti-SARS-CoV-2 effects of two *i*.*p*. dose levels of the mAb cocktail. The three subgroups received *i*.*p*. doses of either a control IgG1 isotype sham (25 mg/Kg), a high dose of mAb cocktail (25 mg/Kg), or a medium dose mAb cocktail (5 mg/Kg). Following treatment, mice were observed for an additional 5 days. During this observation period, all mice received daily clinical health exams by a veterinary technician without knowledge of group assignments. During these exams, clinical symptoms of SARS-CoV-2 were scored, body weight was measured, and determinations of early humane euthanasia were made (see below). At the end of the 5-day observation period, all surviving mice were humanely euthanized. Following euthanasia (either during the 5-day observation period or at the study end point on Day 6), whole blood, nasal turbinate, brain, lung, and kidney were collected from each mouse.

#### Intranasal administration of mAb cocktail in mice infected with the Delta variant of SARS-CoV-2

Mice from Cohort 2 (n = 30) were infected with the Delta variant of SARS-CoV-2 as described above. At 6-hours post inoculation, mice were anesthetized with a combination of ketamine (40 mg/ml), xylazine (2 mg/ml), and atropine (0.06 mg/ml) at a dose of 0.1–0.3 ml per 100 grams of body weight administered intramuscularly. Mice were randomly assigned (using Microsoft Excel random number generator function) to one of two groups: a control group administered an IgG1 isotype sham (5 mg/Kg, n = 14), or a treatment group administered a mAb cocktail dose (5 mg/Kg, n = 16). Once fully anesthetized, each mouse was placed in a supine position and 25 μl of either the IgG1 isotype or mAb cocktail (based on group assignment) was slowly administered dropwise into the nares. The mice were then immediately held upright for 30–60 seconds to allow the treatment to drain back into the sinuses. At 24-hours post infection, 7 isotype-treated mice and 8 mAb-treated mice were sacrificed. The remaining 15 mice were observed for an additional 5 days. During this observation period, all mice received daily clinical health exams where clinical symptoms of SARS-CoV-2 were scored, body weight was measured, and determinations of early humane euthanasia were made (see below). At the end of the 5-day observation period, all surviving mice were humanely euthanized. Following euthanasia (either during the 5-day observation period or at the study end point on Day 6), whole blood, nasal turbinate, brain, lung, and kidney were collected from each mouse.

#### Health exams, clinical score calculation, and determination of early euthanasia

Mice received health exams once per day starting on the day of viral inoculation (Day 0) until Day 6 post viral inoculation. Exams were performed by AFRIMS veterinary staff without knowledge of group assignments. The exam consisted of an evaluation of appetite level, general activity, grooming behaviour, lethargy, and hair coat condition. During an exam, each category was given a score of 0 (normal) to 3 (significant signs of illness), and scores were summed together to generate a single daily clinical score for each mouse. Body weight was also recorded after each exam. Any animal with a daily clinical score of ≥5 or a recorded loss of ≥20% body weight (relative to Day 0 body weight) was determined to meet criteria for early humane euthanasia and was sacrificed immediately.

#### Viral quantification via PCR

For whole blood, lung, and nasal turbinate tissue samples, SARS-CoV-2 RNA was extracted using the QIAamp viral RNA mini kit (QIAGEN, Germany). SARS-CoV-2 real-time quantitative RT-PCR was performed using the SuperScript III Platinum One-Step Quantitative RT-PCR kit (Invitrogen) per the manufacturer’s instructions, using the Applied Biosystems 7500 Fast Real-Time PCR systems (Life Technologies). Limit of quantification of the assay was 5 genome equivalents (GE)/ml.

### Statistical analysis

Statistical analysis was performed using GraphPad Prism 8.0. Group sizes were determined by a power calculation using mortality as the primary outcome measure. Parameters included an estimated 80% mortality in controls, a 90% reduction as an effect of treatment, an alpha of 0.05, and a target power of 80. ANOVA was the preferred test for analysis; however, the data did not meet the necessary normality and equal variance assumptions. Thus, Mann-Whitney t-test (non-parametric) was utilized to determine statistically significant differences between groups. A *P* value less than 0.05 (*P* ≤ 0.05) was considered as statistically significant. All analyses were performed by a laboratory technician without knowledge of group assignments.

## Supporting information

S1 Table X-Ray crystallography refinement data. (DOC)

S2 Table Lists of binding residues of SARS-CoV-2 RBD protein (WT) that formed contact interface with the residues in CDRs of the 1D1 Fab. (DOC)

S3 Table Lists of binding residues of SARS-CoV-2 RBD protein (Beta) that formed contact interface with the residues in CDRs of the 3D2 Fab. (DOC)

## References

[1] Latinne A, Hu B, Olival KJ, Zhu G, Zhang L, Li H, et al. Origin and cross-species transmission of bat coronaviruses in China. Nature Communications. 2020;11(1):4235. doi: 10.1038/s41467-020-17687-3 32843626 PMC7447761

[2] Zhou P, Yang X-L, Wang X-G, Hu B, Zhang L, Zhang W, et al. A pneumonia outbreak associated with a new coronavirus of probable bat origin. Nature. 2020;579(7798):270–3. doi: 10.1038/s41586-020-2012-7 32015507 PMC7095418

[3] Wu JT, Leung K, Leung GM. Nowcasting and forecasting the potential domestic and international spread of the 2019-nCoV outbreak originating in Wuhan, China: a modelling study. Lancet. 2020;395(10225):689–97. doi: 10.1016/S0140-6736(20)30260-9 32014114 PMC7159271

[4] Momtazmanesh S, Ochs HD, Uddin LQ, Perc M, Routes JM, Vieira DN, et al. All together to Fight COVID-19. Am J Trop Med Hyg. 2020;102(6):1181–3. doi: 10.4269/ajtmh.20-0281 32323644 PMC7253116

[5] Shang J, Wan Y, Luo C, Ye G, Geng Q, Auerbach A, et al. Cell entry mechanisms of SARS-CoV-2. Proceedings of the National Academy of Sciences. 2020;117(21):11727–34. doi: 10.1073/pnas.2003138117 32376634 PMC7260975

[6] Zhang J, Xiao T, Cai Y, Chen B. Structure of SARS-CoV-2 spike protein. Current Opinion in Virology. 2021;50:173–82. doi: 10.1016/j.coviro.2021.08.010 34534731 PMC8423807

[7] Walls AC, Park YJ, Tortorici MA, Wall A, McGuire AT, Veesler D. Structure, Function, and Antigenicity of the SARS-CoV-2 Spike Glycoprotein. Cell. 2020;181(2):281–92.e6. doi: 10.1016/j.cell.2020.02.058 32155444 PMC7102599

[8] Li W, Moore MJ, Vasilieva N, Sui J, Wong SK, Berne MA, et al. Angiotensin-converting enzyme 2 is a functional receptor for the SARS coronavirus. Nature. 2003;426(6965):450–4. doi: 10.1038/nature02145 14647384 PMC7095016

[9] Lan J, Ge J, Yu J, Shan S, Zhou H, Fan S, et al. Structure of the SARS-CoV-2 spike receptor-binding domain bound to the ACE2 receptor. Nature. 2020;581(7807):215–20. doi: 10.1038/s41586-020-2180-5 32225176

[10] Shang J, Ye G, Shi K, Wan Y, Luo C, Aihara H, et al. Structural basis of receptor recognition by SARS-CoV-2. Nature. 2020;581(7807):221–4. doi: 10.1038/s41586-020-2179-y 32225175 PMC7328981

[11] Harrison AG, Lin T, Wang P. Mechanisms of SARS-CoV-2 Transmission and Pathogenesis. Trends Immunol. 2020;41(12):1100–15. doi: 10.1016/j.it.2020.10.004 33132005 PMC7556779

[12] Madu IG, Roth SL, Belouzard S, Whittaker GR. Characterization of a highly conserved domain within the severe acute respiratory syndrome coronavirus spike protein S2 domain with characteristics of a viral fusion peptide. J Virol. 2009;83(15):7411–21. doi: 10.1128/JVI.00079-09 19439480 PMC2708636

[13] Walls AC, Tortorici MA, Snijder J, Xiong X, Bosch BJ, Rey FA, et al. Tectonic conformational changes of a coronavirus spike glycoprotein promote membrane fusion. Proc Natl Acad Sci U S A. 2017;114(42):11157–62. doi: 10.1073/pnas.1708727114 29073020 PMC5651768

[14] Structure Li F., Function, and Evolution of Coronavirus Spike Proteins. Annu Rev Virol. 2016;3(1):237–61. 27578435 10.1146/annurev-virology-110615-042301 PMC5457962

[15] Leung NHL. Transmissibility and transmission of respiratory viruses. Nat Rev Microbiol. 2021;19(8):528–45. doi: 10.1038/s41579-021-00535-6 33753932 PMC7982882

[16] Wang J, Du G. COVID-19 may transmit through aerosol. Ir J Med Sci. 2020;189(4):1143–4. doi: 10.1007/s11845-020-02218-2 32212099 PMC7094991

[17] Wang CC, Prather KA, Sznitman J, Jimenez JL, Lakdawala SS, Tufekci Z, et al. Airborne transmission of respiratory viruses. Science. 2021;373(6558). doi: 10.1126/science.abd9149 34446582 PMC8721651

[18] WHO Coronavirus (COVID-19) Dashboard. 2022 [cited 13 September 2022]. In: World Health Organization [Internet]. Available from: https://covid19.who.int/.

[19] Gandhi RT. The Multidimensional Challenge of Treating Coronavirus Disease 2019 (COVID-19): Remdesivir Is a Foot in the Door. Clin Infect Dis. 2021;73(11):e4175–e8. doi: 10.1093/cid/ciaa1132 32735655 PMC7454361

[20] Fischer WA, Eron JJ 2nd, Holman W Jr., Cohen MS, Fang L, Szewczyk LJ, et al. A phase 2a clinical trial of molnupiravir in patients with COVID-19 shows accelerated SARS-CoV-2 RNA clearance and elimination of infectious virus. Sci Transl Med. 2022;14(628):eabl7430. doi: 10.1126/scitranslmed.abl7430 34941423 PMC10763622

[21] Hammond J, Leister-Tebbe H, Gardner A, Abreu P, Bao W, Wisemandle W, et al. Oral Nirmatrelvir for High-Risk, Nonhospitalized Adults with Covid-19. N Engl J Med. 2022;386(15):1397–408. doi: 10.1056/NEJMoa2118542 35172054 PMC8908851

[22] Weinreich DM, Sivapalasingam S, Norton T, Ali S, Gao H, Bhore R, et al. REGEN-COV Antibody Combination and Outcomes in Outpatients with Covid-19. N Engl J Med. 2021;385(23):e81. doi: 10.1056/NEJMoa2108163 34587383 PMC8522800

[23] Gupta A, Gonzalez-Rojas Y, Juarez E, Crespo Casal M, Moya J, Rodrigues Falci D, et al. Effect of Sotrovimab on Hospitalization or Death Among High-risk Patients With Mild to Moderate COVID-19: A Randomized Clinical Trial. Jama. 2022;327(13):1236–46. doi: 10.1001/jama.2022.2832 35285853 PMC8922199

[24] Dougan M, Azizad M, Mocherla B, Gottlieb RL, Chen P, Hebert C, et al. A randomized, placebo-controlled clinical trial of bamlanivimab and etesevimab together in high-risk ambulatory patients with COVID-19 and validation of the prognostic value of persistently high viral load. Clin Infect Dis. 2021. 10.1093/cid/ciab912 PMC9402688 34718468

[25] COVID-19 Treatment Guidelines. 2022 [cited 1 August 2022]. In: National Institutes of Health [Internet]. Available from: https://www.covid19treatmentguidelines.nih.gov/therapies/antiviral-therapy/.

[26] Kim C, Ryu DK, Lee J, Kim YI, Seo JM, Kim YG, et al. A therapeutic neutralizing antibody targeting receptor binding domain of SARS-CoV-2 spike protein. Nat Commun. 2021;12(1):288. doi: 10.1038/s41467-020-20602-5 33436577 PMC7803729

[27] Hingankar N, Deshpande S, Das P, Rizvi ZA, Wibmer CK, Mashilo P, et al. A combination of potently neutralizing monoclonal antibodies isolated from an Indian convalescent donor protects against the SARS-CoV-2 Delta variant. PLoS Pathog. 2022;18(4):e1010465. doi: 10.1371/journal.ppat.1010465 35482816 PMC9089897

[28] Tuccori M, Ferraro S, Convertino I, Cappello E, Valdiserra G, Blandizzi C, et al. Anti-SARS-CoV-2 neutralizing monoclonal antibodies: clinical pipeline. MAbs. 2020;12(1):1854149. doi: 10.1080/19420862.2020.1854149 33319649 PMC7755170

[29] Jones BE, Brown-Augsburger PL, Corbett KS, Westendorf K, Davies J, Cujec TP, et al. LY-CoV555, a rapidly isolated potent neutralizing antibody, provides protection in a non-human primate model of SARS-CoV-2 infection. bioRxiv. 2020.

[30] Mariotti S, Capocefalo A, Chiantore MV, Iacobino A, Teloni R, De Angelis ML, et al. Isolation and Characterization of Mouse Monoclonal Antibodies That Neutralize SARS-CoV-2 and Its Variants of Concern Alpha, Beta, Gamma and Delta by Binding Conformational Epitopes of Glycosylated RBD With High Potency. Front Immunol. 2021;12:750386. doi: 10.3389/fimmu.2021.750386 34764961 PMC8576447

[31] Hansen J, Baum A, Pascal KE, Russo V, Giordano S, Wloga E, et al. Studies in humanized mice and convalescent humans yield a SARS-CoV-2 antibody cocktail. Science. 2020;369(6506):1010–4. doi: 10.1126/science.abd0827 32540901 PMC7299284

[32] Pinto D, Park YJ, Beltramello M, Walls AC, Tortorici MA, Bianchi S, et al. Cross-neutralization of SARS-CoV-2 by a human monoclonal SARS-CoV antibody. Nature. 2020;583(7815):290–5. doi: 10.1038/s41586-020-2349-y 32422645

[33] Emergency Use Authorization. 2020 [cited 1 August 2022]. In: U.S. Food & Drug Administration [Internet]. Available from: https://www.fda.gov/emergency-preparedness-and-response/mcm-legal-regulatory-and-policy-framework/emergency-use-authorization.

[34] Anti-SARS-CoV-2 Antibody Products. 2022 [cited 1 August 2022]. In: National Institutes of Health [Internet]. Available from: https://www.covid19treatmentguidelines.nih.gov/therapies/anti-sars-cov-2-antibody-products/.

[35] Fact sheet for healthcare providers: emergency use authorization for bebtelovimab. 2022 [cited 1 August 2022]. In: Food and Drug Administration [Internet]. Available from: https://www.fda.gov/media/156152/download.

[36] Fact sheet for healthcare providers: emergency use authorization for Evusheld (tixagevimab co-packaged with cilgavimab). 2022 [cited 1 August 2022]. In: Food and Drug Administration [Internet]. Available from: https://www.fda.gov/media/154701/download.

[37] Tegally H, Wilkinson E, Giovanetti M, Iranzadeh A, Fonseca V, Giandhari J, et al. Detection of a SARS-CoV-2 variant of concern in South Africa. Nature. 2021;592(7854):438–43. doi: 10.1038/s41586-021-03402-9 33690265

[38] Leung K, Shum MH, Leung GM, Lam TT, Wu JT. Early transmissibility assessment of the N501Y mutant strains of SARS-CoV-2 in the United Kingdom, October to November 2020. Euro Surveill. 2021;26(1). doi: 10.2807/1560-7917.ES.2020.26.1.2002106 33413740 PMC7791602

[39] Cameroni E, Bowen JE, Rosen LE, Saliba C, Zepeda SK, Culap K, et al. Broadly neutralizing antibodies overcome SARS-CoV-2 Omicron antigenic shift. Nature. 2022;602(7898):664–70. doi: 10.1038/s41586-021-04386-2 35016195 PMC9531318

[40] Liu L, Iketani S, Guo Y, Chan JF, Wang M, Liu L, et al. Striking antibody evasion manifested by the Omicron variant of SARS-CoV-2. Nature. 2022;602(7898):676–81. doi: 10.1038/s41586-021-04388-0 35016198

[41] Tracking SARS-CoV-2 variants. 2022 [cited 2 August 2022]. In: World Health Organization [Internet]. Available from: https://www.who.int/activities/tracking-SARS-CoV-2-variants.

[42] SARS-CoV-2 variant classifications and definitions. 2022 Apr 26 [cited 2 August 2022]. In: Centers for Disease Control and Prevention [Internet]. Available from: https://www.cdc.gov/coronavirus/2019-ncov/variants/variant-classifications.html.

[43] Greaney AJ, Starr TN, Barnes CO, Weisblum Y, Schmidt F, Caskey M, et al. Mapping mutations to the SARS-CoV-2 RBD that escape binding by different classes of antibodies. Nat Commun. 2021;12(1):4196. doi: 10.1038/s41467-021-24435-8 34234131 PMC8263750

[44] Hastie KM, Li H, Bedinger D, Schendel SL, Dennison SM, Li K, et al. Defining variant-resistant epitopes targeted by SARS-CoV-2 antibodies: A global consortium study. Science. 2021;374(6566):472–8. doi: 10.1126/science.abh2315 34554826 PMC9302186

[45] Karim SSA, Karim QA. Omicron SARS-CoV-2 variant: a new chapter in the COVID-19 pandemic. Lancet. 2021;398(10317):2126–8. doi: 10.1016/S0140-6736(21)02758-6 34871545 PMC8640673

[46] Tracking of hCoV-19 Variants. 2021 [cited 2 August 2022]. In: GISAID [Internet]. Available from: https://gisaid.org/hcov19-variants/.

[47] Classification of Omicron (B.1.1.529): SARS-CoV-2 Variant of Concern. 2021 Nov 26 [cited 2 August 2022]. In: World Health Organization [Internet]. Available from: https://www.who.int/news/item/26-11-2021-classification-of-omicron-(b.1.1.529)-sars-cov-2-variant-of-concern.

[48] Elliott P, Bodinier B, Eales O, Wang H, Haw D, Elliott J, et al. Rapid increase in Omicron infections in England during December 2021: REACT-1 study. Science. 2022;375(6587):1406–11. doi: 10.1126/science.abn8347 35133177 PMC8939772

[49] Chen J, Wang R, Gilby NB, Wei GW. Omicron (B.1.1.529): Infectivity, vaccine breakthrough, and antibody resistance. ArXiv. 2021. 34989238 10.1021/acs.jcim.1c01451 PMC8751645

[50] Variants of the Virus. 2021 Aug 11 [cited 3 August 2022]. In: Centers for Disease Control and Prevention [Internet]. Available from: https://www.cdc.gov/coronavirus/2019-ncov/variants/index.html.

[51] Vonrhein C, Flensburg C, Keller P, Sharff A, Smart O, Paciorek W, et al. Data processing and analysis with the autoPROC toolbox. Acta Crystallogr D Biol Crystallogr. 2011;67(Pt 4):293–302. doi: 10.1107/S0907444911007773 21460447 PMC3069744

[52] Kabsch W. XDS. Acta Crystallogr D Biol Crystallogr. 2010;66(Pt 2):125–32. doi: 10.1107/S0907444909047337 20124692 PMC2815665

[53] Evans PR, Murshudov GN. How good are my data and what is the resolution? Acta Crystallogr D Biol Crystallogr. 2013;69(Pt 7):1204–14. doi: 10.1107/S0907444913000061 23793146 PMC3689523

[54] McCoy AJ, Grosse-Kunstleve RW, Adams PD, Winn MD, Storoni LC, Read RJ. Phaser crystallographic software. J Appl Crystallogr. 2007;40(Pt 4):658–74. doi: 10.1107/S0021889807021206 19461840 PMC2483472

[55] Murshudov GN, Skubák P, Lebedev AA, Pannu NS, Steiner RA, Nicholls RA, et al. REFMAC5 for the refinement of macromolecular crystal structures. Acta Crystallogr D Biol Crystallogr. 2011;67(Pt 4):355–67. doi: 10.1107/S0907444911001314 21460454 PMC3069751

[56] Emsley P, Lohkamp B, Scott WG, Cowtan K. Features and development of Coot. Acta Crystallogr D Biol Crystallogr. 2010;66(Pt 4):486–501. doi: 10.1107/S0907444910007493 20383002 PMC2852313

[57] Krug M, Weiss M, Heinemann U, Mueller U. XDSAPP: A graphical user interface for the convenient processing of diffraction data using XDS. Journal of Applied Crystallography. 2012;45.

