# Supplementary material for: Efficacy of the combination of monoclonal antibodies against the SARS-CoV-2 Beta and Delta variants
Source: PLoS One. 2023 May 4;18(5):e0284173. doi: 10.1371/journal.pone.0284173 (PMC10159178; doi:10.1371/journal.pone.0284173)
Supplement: S1 Table — (DOC) [file pone.0284173.s001.doc]

**Supporting information**

**S1 Table. X-Ray crystallography refinement data.**

|  | **1D1 Fab RBD (PDB ID 8BSE)** | **3D2 Fab RBD (Beta variant) (PDB ID 8BSF)** |
| --- | --- | --- |
| Resolution (Å) | 47.44 - 1.90 (1.94 - 1.90) | 48.07 – 2.20 (2.27 – 2.20) |
| Wavelength (Å) | 0.9537 | 0.9762 |
| Space group | P2_1_2_1_2_1_ | C2 |
| Unit cell (Å,°) | a = 56.18 b = 110.02, c = 149.39 | a = 104.69 b = 71.2, c = 121.25 β = 90.04 ° |
| Completeness (%) | 100.0 (100.0) | 98.9 (98.3) |
| No. of observations / unique reflections | 1 005 518 / 73 863 (61 931 / 4 501) | 317 904 / 44 882 (27 850 / 3 839) |
| Redundancy | 13.6 (13.8) | 7.1 (7.2) |
| <I/σ(I)> | 14.8 (1.1) | 13.0 (1.3) |
| CC(1/2) (%) | 99.9 (66.4) | 99.8 (57.5) |
| R_merge_ (I) (%) | 10.9 (263.1) | 9.6 (143.7) |
| R_pim_ (I) (%) | 3.1 (73.0) | 3.9 (62.7) |
| R_model_ (F) (%) | 17.9 (30.5) | 19.6 (31.4) |
| R_free_ (F) (%) | 21.6 (30.6) | 22.9 (30.1) |
| No. of non-hydrogen atoms | 5349 | 4789 |
| No. of water molecules | 499 | 123 |
| **rms deviations from ideal geometry** |  |  |
| Bond lengths (Å) | 0.012 | 0.009 |
| Bond angles (°) | 1.7 | 1.61 |
| Mean B-factor (Å^2^) Chain A, H,L | 53.0, 42.7, 43.1 | 79.8, 53.3, 44.5 |
| Mean B-factor others (Å^2^) | 71.1 | 71.4 |
| Mean B-factor solvent (Å^2^) | 52.9 | 47.8 |
| **Ramachandran plot quality^##^** |  |  |
| Favored regions (%) | 96.6 | 96.4 |
| Allowed regions (%) | 3.4 | 3.4 |
| Outliers (%) | 0 | 0.2 |

Figures in parentheses are for the highest resolution shell. Other relevant quality indicators can be easily extracted from the PDB file header. ^##^Calculated using SARomics local MolProbity serve (1).

**Reference**

1. Chen VB, Arendall WB, 3rd, Headd JJ, Keedy DA, Immormino RM, Kapral GJ, et al. MolProbity: all-atom structure validation for macromolecular crystallography. Acta crystallographica Section D, Biological crystallography. 2010;66(Pt 1):12-21.
