# Supplementary material for: Efficacy of the combination of monoclonal antibodies against the SARS-CoV-2 Beta and Delta variants
Source: PLoS One. 2023 May 4;18(5):e0284173. doi: 10.1371/journal.pone.0284173 (PMC10159178; doi:10.1371/journal.pone.0284173)
Supplement: S2 Table — (DOC) [file pone.0284173.s002.doc]

**Supporting information**

**S2 Table. Lists of binding residues of SARS-CoV-2 RBD protein (WT) that formed contact interface with the residues in CDRs of the 1D1 Fab.**

| **RBD (WT)** | **1D1 Fab** | | |
| --- | --- | --- | --- |
| **Residue** | **Residue** | **Domains** | **Interactive Bond** |
| R403 | D92 | VL-CDR3 | Salt bridge |
| D420 | S56 | VH-CDR2 | H-bond |
| Y421 | S53 | VH-CDR2 | H-bond |
| L455 | Y33 | VH-CDR1 | H-bond |
| R457 | S53 | VH-CDR2 | H-bond |
| S459 | G54 | VH-CDR2 | H-bond |
| N460 | S56 | VH-CDR2 | H-bond |
| Y473 | S31 | VH-CDR1 | H-bond |
| A475 | N32 | VH-CDR1 | H-bond |
| N487 | D102 | VH-CDR3 | H-bond |
| Y489 | R97 | VH-CDR3 | H-bond |
| Y505 | D92 | VL-CDR3 | H-bond |
