# Supplementary material for: Efficacy of the combination of monoclonal antibodies against the SARS-CoV-2 Beta and Delta variants
Source: PLoS One. 2023 May 4;18(5):e0284173. doi: 10.1371/journal.pone.0284173 (PMC10159178; doi:10.1371/journal.pone.0284173)
Supplement: S3 Table — (DOC) [file pone.0284173.s003.doc]

**Supporting information**

**S3 Table. Lists of binding residues of SARS-CoV-2 RBD protein (Beta) that formed contact interface with the residues in CDRs of the 3D2 Fab.**

| **RBD (Beta)** | **3D2 Fab** | | |
| --- | --- | --- | --- |
| **Residue** | **Residue** | **Domains** | **Interactive Bond** |
| R346 | D55 | VL-CDR2 | Salt bridge |
| S373 | S106 | VL-CDR3 | H-bond |
| N439 | T94 | VH-CDR3 | H-bond |
| N440 | T94 | VH-CDR3 | H-bond |
| L441 | Y110 | VL-CDR3 | H-bond |
| K444 | D57 | VL-CDR2 | H-bond |
| P499 | S95 | VH-CDR3 | H-bond |
| T500 | S95 | VH-CDR3 | H-bond |
